# On Quackery

**Published:** 1802-09-01

**Authors:** 


*August*, 9, 1802.

---

### *To the Editors of the Medical and Phyſical Journal.*

GENTLEMEN,

I BEG leave to offer the following hints to the peruſal of your numerous readers; the moſt of whom, I flatter myſelf, will coincide in opinion with me.

Some of your Correſpondents have wrote very ably againſt Quackery, both domeſtic and public, which does them honour; and it behoves every medical practitioner to take a part, in endeavouring to ſuppreſs ſo great an evil.

Moſt of the above worthy characters have aimed chiefly at annihilating the public notorious advertiſing quacks, who are certainly certainly a great disgrace to this nation; and the ruin of many thousand of our fellow subjects; but while they are encouraged by those who style themselves regulars, (as I will point out hereafter) and the revenue receives a source of wealth, I fear little is to be expected towards the total subduing the same.

But there exists another evil which falls little short of quackery, and which is mentioned in the third volume of your valuable miscellany, by Mr. Urwins, where he says as follows:

"There is a class of individuals, which form a large part of the medical practitioners of this country, who, without education, without abilities, nay, sometimes even destitute of common sense, having perhaps for a few years been in the habit of compounding and vending medicines in the shop of a druggist or an apothecary, have the effrontery to deem themselves qualified to practise physic, and enrol themselves in the list of medical professors!"

It may be said their number can be but few, but there is too much reason to join in the sentiments of the above writer; and for this reason: Able assistants are hard to be obtained, as a variety of ways render them more independent and comfortable than the salary generally given for their services, and the confinement they are subjected to; the consequence is, many practitioners will take an apprentice, perhaps one who has acted in the capacity of an errand boy, if somewhat active, or one almost as inferior; of course, without fee or reward, instruct him in as much as is just requisite to serve in the situation of a better, by which he is rendered very dependent for a few years, till he begins to think himself so far enlightened, as to undertake the care of the health of others, for a voyage or two to Greenland; or else, through interest, gets into a situation in one of the Royal Hospitals, as dispenser, by which a little money is got together, with a view of attending a few lectures. Having so done, he thinks himself perfectly qualified to commence his career of practice in the world, where perhaps he is prosperous (not from true knowledge, but chance), others take it as an example, and follow it. Some again, though placed out with adequate means for pursuing studies sufficient to inform the mind for so great an undertaking, neglect it almost totally, and think, that the obtaining a few certificates from the hands of their teachers, who are men of eminence, will be a sufficient sanction for their success; but how much they are deceived, for as real merit cannot afford them the necessary assistance to get forward in life, their sole object is not the welfare of their fellow creature, who seeks, and supposes he is obtaining help in his infirmities under the hands of his apothecary: No, the latter is considering himself, himfelf, by fending plenty of medicine (if it can be called fo) to pay for his attendance; when, let the cafe go how it will, the practitioner is very eafy about it.

Again, forry I am to mention it, there is a great want of an open, liberal opinion of, and conduct towards, each other as medical men; fome, from a narrownefs of principle, others from envy at their better's fuccefs, are ever ready to embrace an opportunity of injuring them in their profeffional avocations; inftead of acting with that candour and generofity towards each other, as become men in fo honourable and dignified a profeffion; whereby they not only would be efteemed in the public favour, but render each other reciprocal pleafure and happinefs.

Great complaint was fome time ago raifed againft the chemifts of this metropolis, interfering with the bufinefs of the apothecary; fuch as making up phyficians prefcriptions, and giving advice to patients, both which I think of very little confequence; for as to the former, for the moft part, they were merely retailed as other drugs, and confequently fhould imagine it fcarce worth an apothecary's while, if in good medical practice, as it muft interfere very much with fuch practice; another thing, it could not recompence him according to the trouble which would be expected from him, fuch as vifiting the patients, perhaps upon the moft trivial circumftance that might arife from the action of the medicine: Now the druggift is not liable to the above, it not being his cuftom; therefore, the prefcriber is only informed of it, and that without any additional trouble to him.

In refpect to the latter, few, I believe, are capable of giving that advice to patients, as to be an object worthy of notice, by keeping them from the regular practitioner in medicine; and may not the above practice have arifen from many apothecaries vending drugs by retail, and fo far interfering with the bufinefs of the chemift? Here it is that Quackery is, in a great meafure, affifted by apothecaries in retailing quack medicines; for may it not appear to the purchafers that they are approved of by the vendors; this I am fure of, that they have recommended them when afked their opinion of fuch and fuch medicines; confequently, Quackery is fanctioned by thofe who ought to be the chief opponents to fo evil a practice.

There are many medical publications, however, well intended by the authors; fome few have contributed, in no fmall degree, to the encouragement of domeftic Quackery; for the natural curiofity of the human mind is fuch, that it is ever fearching after novelty, and thefe books being eafily purchafed, every thing appears plain therein; therefore, not the moft trivial vial complaint ſupervenes, but a remedy is ſought after, without regard to age, ſex, or condition of the patient; the practice is purſued, till imaginary diſeaſe is rendered real, and life is made miſerable for a longer or ſhorter period.

Having pointed out ſome of the evils, on the ſubject of Quackery, in both points of view, its remedy, in my humble opinion, is clearly pointed out in the practice of a contrary plan to the above-mentioned; but the moſt effectual towards raiſing the medical profeſſion to the reſpectability it ſo juſtly deſerves, and ſtamp honor on the practitioners, and at the ſame time afford no room to the ignorant, would be, by the Legiſlature inveſting a certain claſs of members of the Company of Apothecaries, (either by charter, or in any way its wiſdom may ſeem fit, ſimilar to the Royal College of Surgeons,) with a power to examine the fitneſs of all in future, to practice the ſcience of Pharmacy; whereby, only thoſe who have it in their power, would place their ſons in the profeſſion, and who could afford the neceſſary means to enable them to ſtudy, in order to undergo ſuch examination.

At the ſame time, it might appear proper, to obtain of the Legiſlature, ſome act, or power, towards ſuppreſſing a claſs of men, who practice ſo largely in duping the unwary public, (by the bye, many of them do not come under the cognizance of the new act ſo ſtrongly againſt quack medicines) who have the misfortune to fall the victims of a dire diſeaſe, and one which requires all the ſtrength of underſtanding and judgment, to render leſs deſtructive to the human race, viz. ſyphilis, than to be ſuffered to be the means of living for the idle and ignorant, at the expenſe of lives and property of whole families; for as character is no object to them, ſo the greater deception, by promiſing more than poſſible is their greateſt art; for what can be thought of a painter and glazier turning quack doctor, and others equally abſurd, only by changing their name and place of abode?

For fear of obtruding too much on your goodneſs, and taking up too much room in your valuable publication, I will cloſe the ſubject, by ſincerely hoping, that ſome of your much abler Correſpondents will continue the ſubject; and that their efforts may be crowned with all the ſucceſs it ſo much merits, is the firm wiſh of one, who is happy in ſubſcribing himſelf

A Medical Practitioner.

*August* 9, 1802.

*Observations*

